# P-1169. Obeldesivir and GS-441524 Antiviral Activity against L Protein Mutants in Respiratory Syncytial Virus (RSV) Minigenome and Recombinant Infectious Virus Systems

**DOI:** 10.1093/ofid/ofaf695.1362

**Published:** 2026-01-11

**Authors:** Jasmine Moshiri, J Lizbeth Reyes Zamora, Dong Han, Josolyn Chan, Nadine Peinovich, Christopher Richards, Stacey Eng, John P Bilello, Charlotte Hedskog

**Affiliations:** Gilead Sciences, Inc., Foster City, CA; Gilead Sciences, Inc., Foster City, CA; Gilead Sciences, Inc., Foster City, CA; Gilead Sciences, Inc., Foster City, CA; Gilead Sciences, Inc., Foster City, CA; Gilead Sciences, Inc., Foster City, CA; Gilead Sciences, Inc., Foster City, CA; Gilead Sciences, Inc., Foster City, CA; Gilead Sciences, Inc., Foster City, CA

## Abstract

**Background:**

Obeldesivir (ODV), an orally bioavailable prodrug of the GS-441524 nucleoside analog, is under evaluation for treatment of RSV in nonhospitalized patients. Here, we report the antiviral activity of GS-441524 and ODV against RSV L polymerase substitutions that emerged during in vitro resistance selections in the presence of ODV, GS-441524, or ALS-8112 (parent nucleoside of lumicitabine) using an optimized RSV minigenome phenotypic assay and a recombinant infectious virus system.

**Methods:**

In the optimized minigenome assay, plasmids encoding L, N, P, and M2-1 proteins were reverse-transfected with a firefly luciferase reporter into HEK293T cells, with luciferase activity measured at 48 hours. Phenotypic testing in an infectious virus system used HEp-2 cells with a cellomics-based immunofluorescent readout measured at 72 hours. The L polymerase substitutions C319R, I777L, and L1453W identified during in vitro resistance selection with GS-441524 or ODV and the QUAD substitutions (M628L + A789V + L795I + I796V), both alone and in combination, that emerged during in vitro selection with ALS-8112 were tested in both systems. GS-441524 and ODV half-maximal effective concentration (EC_50_) values were determined using variable slope non-linear regression.

**Results:**

L polymerase substitutions C319R, A789V, L795I, I796V, L1453W, and QUAD had similar susceptibility to GS-441524 and ODV as the wild type reference in the minigenome system (< 1.09- and < 1.20-fold change in EC_50_, respectively) and the infectious virus system (< 1.79- and < 2.00-fold change in EC_50_, respectively). The I777L substitution was associated with low-level reduced susceptibility to GS-441524 and ODV in both the minigenome system (2.06- and 2.07-fold change in EC_50_, respectively) and the infectious virus system (3.31- and 3.83-fold change in EC_50_, respectively). The M628L mutant could not be tested due to poor replication. Overall, GS-441524 and ODV EC_50_ fold changes were concordant in both phenotypic systems.

**Conclusion:**

These data support the use of the optimized RSV minigenome system to evaluate the susceptibility of RSV L polymerase substitutions against GS-441524 and ODV and indicate that these drugs have a high barrier to resistance development.

**Disclosures:**

Jasmine Moshiri, PhD, Gilead Sciences, Inc.: Employee|Gilead Sciences, Inc.: Stocks/Bonds (Public Company) J. Lizbeth Reyes Zamora, PhD, Gilead Sciences, Inc.: Employee|Gilead Sciences, Inc.: Stocks/Bonds (Public Company) Dong Han, MS, Gilead Sciences, Inc.: Employee|Gilead Sciences, Inc.: Stocks/Bonds (Public Company) Josolyn Chan, MS, Gilead Sciences, Inc.: Employee|Gilead Sciences, Inc.: Stocks/Bonds (Public Company) Nadine Peinovich, MPH, Gilead Sciences, Inc.: Employee|Gilead Sciences, Inc.: Stocks/Bonds (Public Company) Christopher Richards, PhD, Gilead Sciences, Inc.: Employee|Gilead Sciences, Inc.: Stocks/Bonds (Public Company) Stacey Eng, BS, Gilead Sciences, Inc.: Employee|Gilead Sciences, Inc.: Stocks/Bonds (Public Company) John P. Bilello, PhD, Gilead Sciences, Inc.: Employee|Gilead Sciences, Inc.: Stocks/Bonds (Public Company) Charlotte Hedskog, PhD, Gilead Sciences, Inc.: Employee|Gilead Sciences, Inc.: Stocks/Bonds (Public Company)

